# Transradial ‘combined catheter’ technique for six-vessel cerebral angiography

**DOI:** 10.3389/fneur.2025.1688623

**Published:** 2026-01-13

**Authors:** Rundong Chen, Guoli Duan, Qian Zheng, Guanghao Zhang, Yiban Fang, Pengfei Yang, Yi Xu, Jianmin Liu, Qiang Li

**Affiliations:** 1 Changhai Hospital, Second Military Medical University, Shanghai, China; 2 Shanghai Fourth People's Hospital, Shanghai, China

**Keywords:** transradial access, selective cerebral angiography, combined catheter technique, safety, feasibility

## Abstract

**Background:**

Compared with transfemoral access (TFA), transradial access (TRA) has been widely used in neurointervention, as it has a relatively low rate of access-site complications. However, the method still has several restrictions, particularly with regard to selective angiography. This study aimed to evaluate the technical feasibility and procedural performance of a combined catheter technique designed to facilitate complete six-vessel selective cerebral angiography via TRA.

**Methods:**

We prospectively studied patients who underwent TRA selective cerebral angiography with the combined catheter technique between January 2023 and September 2023. Clinical characteristics, procedural details, complications, and outcomes at discharge were collected. A descriptive analysis was performed.

**Results:**

A total of 48 patients underwent TRA angiography using the combined catheter technique, 15 of whom were male. The median age of the patients was 54.81 ± 13.62 years (30–82). The intermediate or distal catheter types used in patients were Tethys in 27 cases, Envoy DAXB in 6 cases, Envoy DA in 6 cases, and Navien in 9 cases. All patients successfully received selective six-vessel angiography, including bilateral internal carotid arteries (ICAs), external carotid arteries (ECAs), and vertebral arteries (VAs), and completed the follow-up neurointervention procedure. Image quality scores for the arterial phase, capillary apparatus, and venous phase were all approximately 15, which indicates that all patients showed superior visualization of the vasculature. Average selection times per vessel were 230.50 ± 72.80 s (124–395).

**Conclusion:**

In this small single-center feasibility series, the combined catheter technique for six-vessel TRA cerebral angiography appeared technically feasible and safe, but these preliminary findings require confirmation in larger comparative studies.

## Introduction

Transfemoral access (TFA) is the traditional access for neurointervention and allows high-quality imaging of intracranial vessels. However, several access-site complications have been reported, including local ecchymosis, severe hemorrhage, hemorrhagic shock, femoral artery pseudoaneurysm, femoral arteriovenous fistula, and femoral artery occlusion (1, 2).

Transradial access (TRA) has been widely used in cerebral angiography and endovascular procedures because the cardiology literature has revealed that there was a 60% reduction in access-site complications compared with TFA, leading to a “radial-first strategy” (3–5). Similar safety benefits and enhanced patient preferences have also been observed with neurointerventional practice (6–8).

Acquiring distal access and performing selective angiography via TRA, however, require a long learning curve because this is a new technique (7, 9). In our high-volume radial practice, we observed that conventional single-catheter approaches, typically using a Simmons or similar diagnostic catheter, often struggle to achieve stable purchase for selective catheterization of the left internal carotid arteries (LICA), left vertebral arteries (LVA), and ECAs, especially in patients with type II/III aortic arches or steep great-vessel take-offs. Some lesions may be missed by non-selective angiography or require conversion to TFA procedures due to difficulty in establishing access via TRA. In addition, repeated wire and catheter exchanges, as well as the deep advancement of stiff wires into intracranial segments—sometimes required to support catheter advancement—can prolong procedural time, increase radial artery spasms, and theoretically increase the risk of vessel injury. To address these specific limitations within the TRA paradigm, we developed a combined catheter technique for TRA cerebral angiography, consisting of a 5F Simmon-2 catheter nested inside a 6F distal access catheter (DAC). The Simmons component provides robust aortic arch support, while the softer DAC can be advanced distally under this support to facilitate complete six-vessel selective angiography, including ECAs and the contralateral VA, with fewer exchanges and potentially greater stability.

This study evaluated the technical feasibility, efficiency, and safety of a combined catheter technique designed to facilitate full six-vessel angiography via TRA. We hypothesized that, in a prospective single-center series, this combined-catheter strategy would achieve a high rate of complete six-vessel selective angiography with diagnostic image quality, acceptable vessel selection times, and a low incidence of access-site and systemic complications.

## Methods

We conducted a prospective, single-center, single-arm observational study primarily aimed at evaluating the technical feasibility of a combined-catheter strategy for six-vessel TRA cerebral angiography. We enrolled 48 consecutive adult patients who underwent TRA selective cerebral angiography with a combined catheter technique between January 2023 and September 2023. All patients provided informed consent before the procedure. Institutional review board approval was obtained from our hospital. Patients were included if they underwent elective diagnostic cerebral angiography via right TRA. The exclusion criteria included the Barbeau Type D waveform, ipsilateral AV fistula, radial artery occlusion, severe forearm deformity, or very small-caliber radial artery on physical examination. Routine preprocedural ultrasound measurement of radial artery diameter was not performed; instead, radial suitability for a 6F sheath was assessed clinically using radial pulse palpation, blood pressure measurement, and Barbeau waveform testing. Patients deemed unsuitable for a 6F radial sheath were directed to alternative access routes and were not enrolled in this series. All procedures were performed by two senior neurointerventionists (high-volume TRA operators, each with >500 prior TRA cases). Clinical characteristics, procedural details, complications, and outcomes at discharge were collected.

The primary outcome of this feasibility study was the rate of complete six-vessel cerebral angiographic success; secondary outcomes included vessel selection time, image-quality scores, and the incidence of access-site or systemic complications. In this study, “six-vessel cerebral angiography” was defined as diagnostic-quality selective catheterization of the left and right internal carotid arteries (LICA, RICA), the left and right external carotid arteries (LECA, RECA), and the left and right vertebral arteries (LVA, RVA) within a single session. “Complete success” was prospectively defined as achieving diagnostic-quality selective catheterization of all six vessels in the same TRA session without conversion to an alternative access route.

Image-quality scoring. Image quality was graded, adapted from Söderman et al. (6) using three angiographic phases—arterial, capillary/parenchymal, and venous—each scored on a 5-point Likert scale (1 = non-diagnostic, 2 = poor, 3 = adequate, 4 = good, and 5 = excellent). The sum score ranged from 3 to 15. For this study, diagnostic quality required a sum ≥12 with no phase <4; “superior visualization” denotes 15 of 15 (i.e., 5 of 5 in all phases). All angiograms were independently scored by two experienced neurointerventionists; in cases of discrepancy in phase scores or overall diagnostic classification, a third senior neurointerventionist adjudicated the final score by consensus.

Given the prospective single-arm design, only descriptive statistics were used. Continuous variables are presented as mean ± standard deviation, and categorical variables as counts and percentages; no formal hypothesis testing against a control group was performed.

## Description of technique

**Figure 1:**
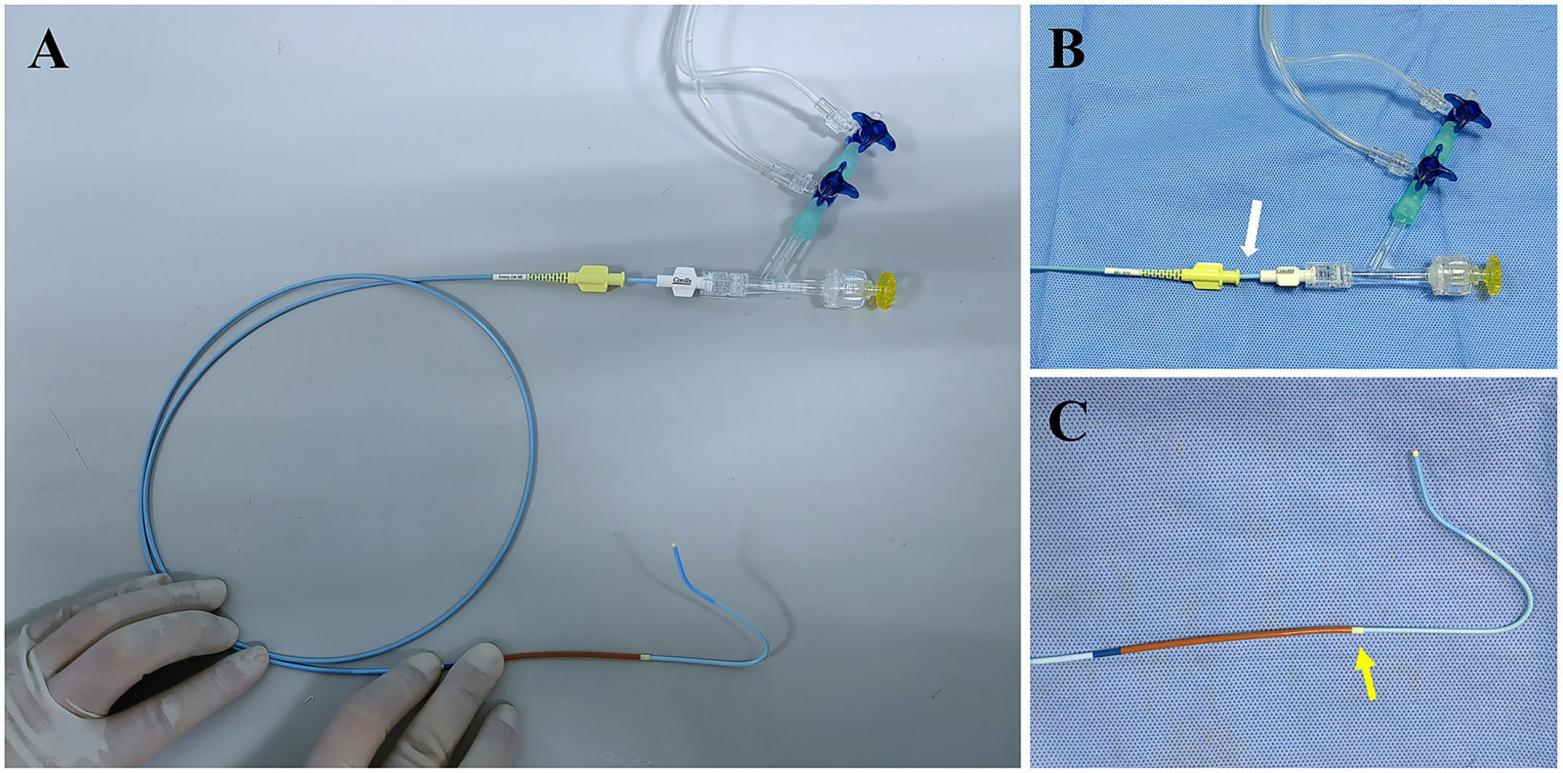
Assembly of the combined catheter system. **(A)** Overall view of the assembled system. **(B)** Enlarged view showing the end of the distal access catheter (white arrow). **(C)** Enlarged view showing the tip of the distal access catheter (yellow arrow).

**Figure 2:**
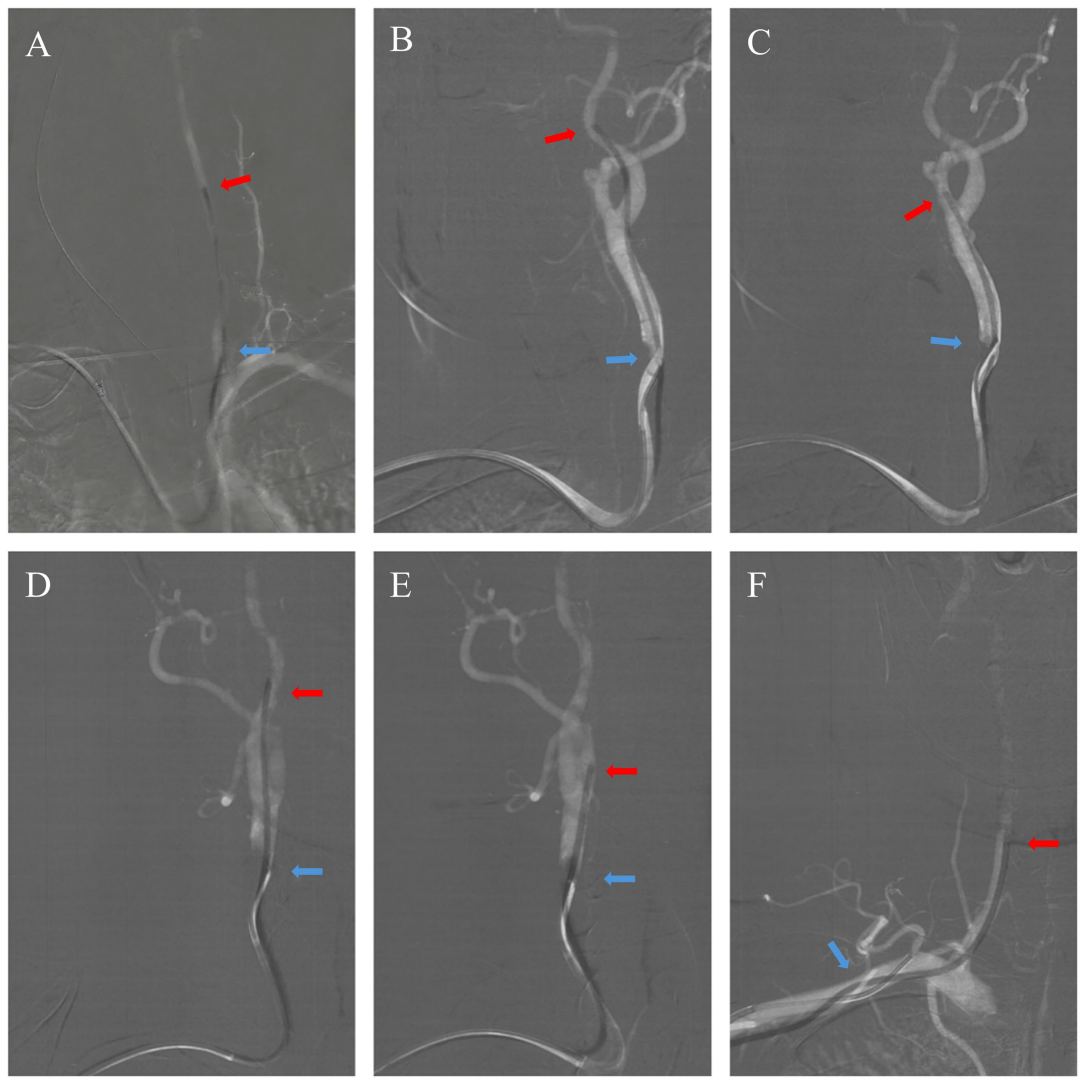
Selective six-vessel cerebral angiography using the combined catheter technique. Panels **(A–F)** correspond to selective angiography of the left vertebral artery (LVA), left internal carotid artery (LICA), left external carotid artery (LECA), right internal carotid artery (RICA), right external carotid artery (RECA), and right vertebral artery (RVA), respectively. The red arrow marks the tip of the distal access catheter (DAC), and the blue arrow marks the tip of the Simmon-2 catheter in each panel.

1. All patients underwent bilateral blood pressure measurement and a Barbeau test before the procedure. The right radial artery was punctured by the standard Seldinger method after being infiltrated with 2% lidocaine. Fluoroscopy showed that the wire was advanced into the radial and brachial arteries. A 16-cm 6F hydrophilic coating sheath (TERUMO, Tokyo, Japan) was also inserted, followed by infusing a 10-mL “Cocktail” through the lateral tube of the sheath to minimize the risk of vasospasm and thrombosis of the radial artery. The “Cocktail” included 200 μg nitroglycerin, 5,000 IU heparin, and 2.5 mg verapamil. Radial patency was documented before the procedure (which required only Barbeau types A–C but not type D) and re-checked 2 h after removing the compression device using a reverse Barbeau maneuver.
2. The catheters were connected to a heparinized saline drip via a hemostasis valve and three-way stopcock, ensuring that the air in the lumen was evacuated and air embolism was prevented. A 125-cm 5F Simmon-2 catheter (Cordis, Florida, USA) was put into a 6F DAC [Envoy DA, Envoy DA XB (Codman, Massachusetts, USA)]; Navien (Medtronic, Minnesota, USA); and Tethys (Peijia, Suzhou, China), which was referred to as a combined catheter. We typically used a 105-cm DAC, which guaranteed the exposure of the 13-cm tip of the Simmon-2 catheter after the assembly of the combined catheters (Figure 1). During inner-catheter manipulations, the Simmon-2 catheter (nested within the distal-access catheter) was maintained on a slow heparinized-saline drip via a three-way stopcock; no continuous positive-pressure flush was applied to the outer distal-access catheter (the DAC used lacked a proximal hemostasis valve/side-arm for continuous infusion).
3. The technique of combined catheter
  - The combined catheters were advanced into the aortic arch via the right subclavian artery and the innominate artery over a hydrophilic wire (TERUMO Tokyo, Japan). Then, we reconstituted the reverse curve of the Simmon-2 catheter. Reconstitution in the descending aorta was preferred over the aortic valve to avoid the risk of cardiac arrhythmias caused by a catheter or wire misdirected into the ventricle.
  - First, a patient with normal anatomy would normally receive the cerebral angiogram of the vessel of interest. The left vertebral artery (LVA) angiogram would be performed first, as it was the easiest way to navigate from left to right. The wire could often be directed into the LVA on the roadmap through rotating, pushing, or pulling the Simmon-2 catheter to select the left subclavian artery. The DAC was then advanced over the wire, allowing for selective catheterization of LVA, while the Simmon-2 catheter was fixed *in situ* (Figure 2).
  - After performing the LVA angiogram, the 6F DAC needs to be retracted back over the Simmon-2 catheter so that the reverse tip can maintain its original shape. The Simmon-2 catheter was then navigated into the left common carotid artery (LCCA), followed by selective catheterization of the left internal carotid artery (LICA) and the external carotid artery (LECA) using the same DAC-guided technique described in step B.
  - Angiography of the right internal carotid artery (RICA), the right external carotid artery (RECA), and the right vertebral artery (RVA) was performed sequentially. In particular, right vertebral angiography may optionally use a Simmon-2 catheter with or without a distal approach catheter. After the standard six-vessel cerebral angiogram was completed, the Simmon-2 catheter was withdrawn into the DAC and subsequently removed. The DAC could be used to complete aneurysm embolization, stent implantation, or other treatments.
4. Patent hemostasis: After the sheath was removed, hemostasis of the radial artery could be achieved using a radial artery compression hemostasis device. The device’s pressure was achieved based on the results of the “reverse Barbeau test” (compressing the ulnar artery manually with a pulse oximeter attached to the patient’s index finger and monitoring the pulse wave). This allowed the radial artery to remain open with minimal pressure required by the compression device.
5. Post-angiogram management: The puncture site and color of the extremities were checked. The compression device was used 2 h after the procedure. A “reverse Barbeau test” was performed to determine whether the radial artery was patent.

## Results

A total of 48 patients performed TRA angiography using the combined catheter technique, 15 of whom were male. The median age of the patients was 54.81 ± 13.62 years (30–82). Among them, patients could be divided into the following types with different symptoms: 33 patients with intracranial aneurysms, 6 patients with intracranial artery stenosis, 1 patient with middle cerebral artery dolichoectasia, 3 patients with acute ischemic stroke, and 5 patients with arteriovenous malformations. Patients diagnosed with diverse aortic arch types could be classified as follows: 21 patients were type II, 18 patients were type I, and the other 9 patients were type III. The types of intermediate catheter, or DAC, used on patients could be categorized as follows: Tethys in 27 cases, Envoy DAXB in 6 cases, Envoy DA in 6 cases, and Navien in 9 cases. Complete procedural success was achieved in all 48 procedures (100%). No procedure required crossover to transfemoral access (0 of 48).

All of the patients had successfully performed selective six-vessel angiography, including bilateral ICAs, ECAs, and VAs, and completed the follow-up neurointervention procedure. Image quality score (3–15 composite) was 15 in all cases (5 of 5 across arterial, capillary/parenchymal, and venous phases), indicating superior visualization in every patient. Mean vessel selection time (from DAC positioning to successful contrast injection) was 230.5 ± 72.8 s. No access-site complications or systemic complications were observed (Table 1).

**Table 1:** Clinical characteristics, procedural details, complications, and outcomes of patients.

Variable	*N* = 48
Age (yr,) [range]	54.81 ± 13.62 (30–82)
Sex	
Male	15 (31.25%)
Female	33 (68.75%)
Types of aortic arch	
I	21 (43.75%)
II	18 (37.5%)
III	9 (18.75%)
Intermediate catheter or distal access catheter	
Tethys	27 (56.25%)
Envoy DAXB	6 (12.50%)
Envoy DA	6 (12.50%)
Navien	9 (18.75%)
Disease	
Aneurysm	33 (68.75%)
Stenosis	6 (12.50%)
Dolichoectasia	1 (2.08%)
Acute ischemic stroke	3 (6.25%)
Arteriovenous malformation	5 (10.42%)
Image quality score	15
Average selection time per vessel (s,) [range]	230.50 ± 72.80 (124–395)
Access-site or systemic complications	0 (0%)

## Discussion

Interventional cardiologists with more than 20 years of experience have demonstrated that TRA has fewer access-site complications, shorter hospital stays, lower hospital costs, and improved patient satisfaction compared with transfemoral access (7). Nonetheless, the access site for selective cerebral angiography has not yet shifted to radial-first (8), largely because TRA still has several technical difficulties and limitations.

After completing 1,000 of selective TRA cerebral angiographies, we found that the Simmons 2 catheter offered the broadest ability to access the great vessels, as suggested by other researchers (9). However, it was not easy to perform selective angiography, especially of the LICA, LVA, and ECA (10), as the Simmons 2 catheter reverse curve exists, which prevents the catheter from changing shape effortlessly (11). Initially, we could only perform angiography of the common carotid arteries (CCAs) and SAs, but this sacrificed the quality of the image and even made it impossible to accurately evaluate the condition. Thus, we had to turn to the femoral artery approach, which increased the patient’s discomfort. In later cases, a 0.035-in, 260-cm extra-stiff wire (RF*PA35263M, TERUMO, Tokyo, Japan) was used to access the ICA in some patients (12), which meant that the wire needed to be passed into the cavernous segment (C4) or higher to provide adequate support for catheter advancement. Through this approach, the artery and the catheter curve could be straightened, facilitating smooth passage of the catheter into the ICA (13). However, the extra-stiff wire was difficult to control, as it often moved back and forth quickly. During the surgery, the wire occasionally entered intracranial blood vessels, posing a risk of vessel dissection. Moreover, repeated rotation of the catheter could even lead to twisting or even fracture of the catheter.

In addition, wire exchanges and repeated selection attempts required more time, increasing the potential risk of thrombotic events and radial artery spasms, as well as significantly prolonging the procedure time. We could overcome most of these limitations using the combined catheter technique, which involves a 6F DAC lined with a 125-cm 5F Simmon-2 catheter. The Simmon-2 catheter has extraordinary support due to the relatively stiff tip with the reverse curve. In contrast, the distal part of the DAC was relatively soft to navigate to the distal vessels under the guidance and support of the Simmon-2 catheter without kicking back the catheter by straightening the reverse curve of the Simmon-2 catheter. This technique combined the advantages of both the Simmon-2 catheter to provide support in the aortic arch and the DAC to perform subsequent selective angiography.

Previous studies have reported that the success rates in the right vertebral artery, right internal carotid artery, left internal carotid artery, and left vertebral artery were, respectively, 96.1, 98.6, 82.6, and 52.2%, often excluding ECA selection (14). However, most retrospective analyses excluded patients who required the selection of the external carotid artery (15). More recent neurointerventional series and meta-analyses comparing TRA and TFA for diagnostic cerebral angiography and endovascular procedures have similarly shown comparable procedural efficacy with lower access-site complication rates for TRA (15–17), but these reports have typically used 4–5F diagnostic catheters and have not mandated complete six-vessel protocols or systematic ECA and contralateral VA selection. Therefore, a majority of the studies on the comparison between TRA and TFA show that there were no apparent advantages or disadvantages in cerebral angiography. However, these comparative data are largely derived from retrospective series with operator-selected access routes, frequently using 4–5F catheters and incomplete six-vessel protocols, and they provide limited information about the completeness and efficiency of truly selective six-vessel angiography via TRA, particularly with respect to ECA and contralateral VA selection. There was no further discussion on selective angiography at present. In this study, patients could quickly complete the selection of all intracranial vessels and establish stable distal access, including external carotid arteriography and contralateral VA, which differs from previous studies. The efficiency of this technique is suggested by the mean vessel selection time of 230.50 s, which appeared shorter than the times reported in previous series using conventional approaches (16). This is a descriptive, cross-study comparison, and no direct statistical testing was performed. Our findings should therefore be interpreted as complementary to, rather than a replacement for, existing TRA–TFA comparative studies by specifically addressing the technical feasibility of complete six-vessel angiography within a radial-first framework. Large comparative studies and meta-analyses have already shown that TRA and TFA have similar procedural efficacy, with lower access-site complication rates for TRA; our study, therefore, focuses on refining the TRA technique itself rather than re-comparing access routes. Radial artery spasm, a complication of concern for most TRA surgeons, was not observed in this study because exchanging and repeatedly manipulating the catheter were avoided while using the combined catheter technique.

At present, a complete set of combined catheter systems is used worldwide for transradial access interventional therapy, including the Rist catheter system. The use of combined catheters is expected to increase in clinical practice in the future. Based on combined catheter technology, it remains highly significant to combine the Simmon-2 catheter with an intermediate catheter or DAC that offers different advantages, enhancing technical versatility and adaptability. This technique also has its limitations. There was no hemostasis valve or continuous positive-pressure saline infusion at the proximal end of the DAC, so blood and diluted contrast within the coaxial system may theoretically stagnate in the annular space between the 5F and 6F catheters and form thrombus, with a potential risk of distal embolism during catheter manipulation and withdrawal. In practice, we occasionally observed a small, slow, outward seepage of blood–contrast mixture from the proximal hub (on the order of approximately 3 mL) during angiographic runs; this leakage was consistently directed from the catheter lumen toward the outside at the hub rather than toward the distal circulation, and no unexpected thromboembolic events occurred in this series. To minimize the risk of air entrapment and thrombosis in the annular space, the outer DAC was first filled with heparinized saline before the insertion of the Simmons catheter, and patients underwent systemic heparinization according to institutional practice, with careful aspiration and flushing of the inner catheter before each injection. These considerations also highlight the need for the development and clinical use of dedicated unitized transradial catheter systems with integrated hemostasis valves and separate flush lumens. It was believed that there would be more special transradial instruments and skills in the future to further improve the safety and prognosis of neurointerventions.

## Limitations

This study has several limitations. First, it was a single-center, prospective observational series with a small sample size (*n* = 48) and no predefined sample size or statistical power calculation; therefore, the results should be interpreted as hypothesis-generating rather than definitive evidence of safety and efficacy. Second, there was no concurrent control group, and all procedures were performed by high-volume TRA operators, which may limit generalizability to centers with less experience. Third, only descriptive statistics were used, and no formal inferential testing or literature-based statistical comparison was performed. Given the small sample size, boundary estimates (100% six-vessel success and 0 complications), and substantial heterogeneity between our protocol—which mandated systematic six-vessel selective angiography in every patient—and prior TRA/TFA series, we felt that constructing pseudo-control groups from historical summary data would be methodologically problematic and potentially misleading. Any references to previously published TFA or TRA cohorts are therefore qualitative and exploratory, and our findings should not be interpreted as providing definitive statistical evidence of superiority over other access strategies. Moreover, fluoroscopy time, total contrast volume, and total procedure duration were not systematically recorded in this feasibility series; as a result, we used per-vessel selection time as a surrogate measure of procedural efficiency, and future studies should incorporate the standardized collection of these additional metrics to enable more granular comparisons. Finally, we did not perform a systematic long-term follow-up of radial artery patency or delayed complications. Future studies should also systematically compare the combined catheter technique with conventional TRA six-vessel cerebral angiography in terms of procedure time, image-quality scores, and vessel-selection success rates to provide a more comprehensive assessment, ideally in larger, multicenter randomized or prospective controlled studies that include concurrent comparator arms with predefined clinical and imaging endpoints.

## Conclusion

This study suggests that the combined catheter technique is technically feasible for six-vessel TRA cerebral angiography and was not associated with access-site or systemic complications in this small, single-center feasibility cohort. Given the non-randomized design and limited sample size, these results should be interpreted as preliminary and exploratory, providing a hypothesis-generating signal rather than definitive evidence of safety or efficacy. Further studies with larger cohorts, predefined sample sizes and power calculations, and comparative designs are warranted to validate these findings, better define safety, and assess long-term outcomes.

## Data Availability

The raw data supporting the conclusions of this article will be made available by the authors, without undue reservation.

## References

[1] Stone PA Campbell JE Aburahma AF. Femoral pseudoaneurysms after percutaneous access. J Vasc Surg. (2014) 60:1359–66. doi: 10.1016/j.jvs.2014.07.035, 25175631

[2] Stolt M Braun-Dullaeus R Herold J. Do not underestimate the femoral pseudoaneurysm. Vasa. (2018) 47:177–85. doi: 10.1024/0301-1526/a000691, 29439611

[3] Jolly SS Yusuf S Cairns J Niemelä K Xavier D Widimsky P . Radial versus femoral access for coronary angiography and intervention in patients with acute coronary syndromes (rival): a randomised, parallel group, multicentre trial. Lancet. (2011) 377:1409–20. doi: 10.1016/s0140-6736(11)60404-2, 21470671

[4] Beijk MAM. Transradial access in chronic anticoagulated patients: one step closer to a "radial-first" strategy in all patients. Int J Cardiol. (2022) 348:45–6. doi: 10.1016/j.ijcard.2021.12.011, 34915077

[5] Valgimigli M Gagnor A Calabró P Frigoli E Leonardi S Zaro T . Radial versus femoral access in patients with acute coronary syndromes undergoing invasive management: a randomised multicentre trial. Lancet. (2015) 385:2465–76. doi: 10.1016/s0140-6736(15)60292-6, 25791214

[6] Söderman M Holmin S Andersson T Palmgren C Babić D Hoornaert B. Image noise reduction algorithm for digital subtraction angiography: clinical results. Radiology. (2013) 269:553–60. doi: 10.1148/radiol.13121262, 23737536

[7] Mason PJ Shah B Tamis-Holland JE Bittl JA Cohen MG Safirstein J . An update on radial artery access and best practices for transradial coronary angiography and intervention in acute coronary syndrome: a scientific statement from the American Heart Association. Circ Cardiovasc Interv. (2018) 11:e000035. doi: 10.1161/hcv.0000000000000035, 30354598

[8] Sheriff FG Rodriguez GJ Gupta V Maud A. Letter regarding 'radial first or patient first: a case series and meta-analysis of transradial (Tra) versus transfemoral (Tfa) access for acute ischemic stroke intervention'. J Neurointerv Surg. (2021) 13:e16. doi: 10.1136/neurintsurg-2021-017655, 33906939

[9] Choi DH Yoo CJ Park CW Kim MJ. Four French sheath-based transradial cerebral angiographies in the elderly: a single neurointerventionalist's experience. Interv Neuroradiol. (2022) 29:15910199221083102. doi: 10.1177/15910199221083102, 35234062 PMC10369113

[10] Sweid A Das S Weinberg JH E l Naamani K Kim J Curtis D . Transradial approach for diagnostic cerebral angiograms in the elderly: a comparative observational study. J Neurointerv Surg. (2020) 12:1235–41. doi: 10.1136/neurintsurg-2020-016140, 32769110

[11] Snelling BM Sur S Shah SS Khandelwal P Caplan J Haniff R . Transradial cerebral angiography: techniques and outcomes. J Neurointerv Surg. (2018) 10:874–81. doi: 10.1136/neurintsurg-2017-013584, 29311120

[12] Chen SH Suazo R Saini V Abecassis IJ Yavagal D Starke RM . Radial artery access for cerebral angiography: 2-dimensional operative video. Oper Neurosurg. (2021) 20:E431–e2. doi: 10.1093/ons/opab071, 33861323

[13] Sattur MG Almallouhi E Lena JR Spiotta AM. Illustrated guide to the transradial approach for neuroendovascular surgery: a step-by-step description gleaned from over 500 cases at an early adopter single center. Oper Neurosurg. (2020) 19:181–9. doi: 10.1093/ons/opaa153, 32511707

[14] Jo KW Park SM Kim SD Kim SR Baik MW Kim YW. Is transradial cerebral angiography feasible and safe? A single center's experience. J Korean Neurosurg Soc. (2010) 47:332–7. doi: 10.3340/jkns.2010.47.5.332, 20539791 PMC2883052

[15] Ge B Wei Y. Comparison of transfemoral cerebral angiography and transradial cerebral angiography following a shift in practice during four years at a single center in China. Med Sci Monit. (2020) 26:e921631. doi: 10.12659/msm.921631, 32210222 PMC7115118

[16] Bhatia K Guest W Lee H Klostranec J Kortman H Orru E . Radial vs. femoral artery access for procedural success in diagnostic cerebral angiography: a randomized clinical trial. Clin Neuroradiol. (2021) 31:1083–91. doi: 10.1007/s00062-020-00984-1, 33373017

[17] Tso MK Rajah GB Dossani RH Meyer MJ McPheeters MJ Vakharia K . Learning curves for transradial access versus transfemoral access in diagnostic cerebral angiography: a case series. J Neurointerv Surg. (2022) 14:174–8. doi: 10.1136/neurintsurg-2021-017460, 34078647

